# Retraction: Anti-inflammatory potency of *Locusta migratoria manilensis* cyclopeptides in mast cells and macrophages

**DOI:** 10.1039/d2ra90039d

**Published:** 2022-04-27

**Authors:** Laura Fisher

**Affiliations:** Royal Society of Chemistry Thomas Graham House, Science Park, Milton Road Cambridge CB4 0WF UK advances-rsc@rsc.org

## Abstract

Retraction of ‘Anti-inflammatory potency of *Locusta migratoria manilensis* cyclopeptides in mast cells and macrophages’ by Jie Liu *et al.*, *RSC Adv.*, 2019, **9**, 31296–31305, https://doi.org/10.1039/C9RA06284J.

The Royal Society of Chemistry hereby wholly retracts this *RSC Advances* article. There is a significant amount of unattributed text overlap with a number of articles by different author groups that were not cited in this article, including ref. 1–4 in the Introduction, ref. 1, 2 and 5 in the Results and ref. 1 and 6 in the Discussion sections.

In addition, the raw data provided by the authors could not be used as verifiable raw data to validate the published western blot images.

The considerable amount of unattributed text overlap, and lack of verifiable raw data, undermines the integrity and reliability of the entire article.

Jie Liu agrees to the retraction. The other authors have been informed but have not responded to any correspondence regarding the retraction.

Signed: Laura Fisher, Executive Editor, *RSC Advances*

Date: 29th March 2022

## Supplementary Material
